# Integrated analysis reveals the dysfunction of signaling pathways in uveal melanoma

**DOI:** 10.1186/s12885-022-09822-8

**Published:** 2022-07-05

**Authors:** Songlin Sun, Boxia Guo, Liang Xu, Rui Shi

**Affiliations:** 1 Department of Ophthalmology, Yuncheng Central Hospital, Shanxi Medical University, Yuncheng, Shanxi Province China; 2 Department of Cardiology, Yuncheng Central Hospital, Shanxi Medical University, Yuncheng, Shanxi Province China; 3 Research Center for Translational Medicine, East Hospital, Tongji University School of Medicine, No.150 Jimo Road, Shanghai, 200120 China; 4 Department of Obstetrics and Gynecology, East Hospital, Tongji University School of Medicine, No.1800 Yuntai Road, Shanghai, 200124 China

**Keywords:** Uveal melanoma, Single-cell RNA sequencing, Molecular mechanism, Heterogeneity, Prognosis

## Abstract

**Background:**

Uveal melanoma (UM) is the most common primary intraocular malignancy with a strong tendency to metastasize. The prognosis is poor once metastasis occurs. The treatment remains challenging for metastatic UM, even though our understanding of UM has advanced, mostly because the complexity of the genetic and immunologic background has not been fully explored.

**Methods:**

Single-cell sequencing data were acquired from a healthy dataset and three UM datasets. The differentially expressed genes between primary and metastatic UM in The Cancer Genome Atlas (TCGA) data were attributed to specific cell types and explained with functional annotation. The analysis for cell–cell communication was conducted by “CellChat” to understand the cell crosstalk among the cell clusters and to delineate the dysfunctional signaling pathways in metastatic UM. CCK-8, EdU and transwell assays were performed to verify the function of the genes of interest.

**Results:**

We revealed aberrant signaling pathways with distinct functional statuses between primary and metastatic UM by integrating multiple datasets. The crucial signals contributing most to outgoing or incoming signaling of metastasis were identified to uncover the potential targeting genes. The association of these genes with disease risk was estimated based on survival data from TCGA. The key genes associated with proliferation and metastasis were verified.

**Conclusions:**

Conclusively, we discovered the potential key signals for occurrence and metastasis of UM and provided a theoretical basis for potential clinical application.

**Supplementary Information:**

The online version contains supplementary material available at 10.1186/s12885-022-09822-8.

## Introduction

Uveal melanoma (UM) is a rare disease that arises from melanocytes in the uvea [1]. As a common intraocular malignancy, the annual incidence rate of UM is 4.3 cases per one million people [1, 2]. Half of patients with UM will develop metastatic disease despite treatment of the primary tumor. The liver is the most common metastatic site, followed by the lungs, bone, and skin. No effective therapies are available to prevent the development of metastases. The average survival period for patients suffering from metastatic UM is no more than 1 year.

Genetic risk factors associated with UM disease include mutations in GNAQ and GNA11 [3]. Over 90% of UM patients carry constitutively active mutations in GNAQ and GNA11, which encode the ɑ-subunits Gq and G11 [4]. Mutations in BRCA-associated protein 1 (BAP1) are observed in over 80% of all UMs, and approximately 28% of patients with germline BAP1 alterations will develop a UM and usually result in metastasis within 5 years [5]. In addition, mutations in SF3B1, SRSF2, EIF1AX, and other known cancer genes are observed in UM patients [6].

Intratumoural heterogeneity is regarded as one of the leading factors that determines metastasis, therapeutic resistance and recurrence. UM tissue is not a homogeneous structure but a complex ecosystem, where tumor cells and diverse cell types engage in dynamic crosstalk that leads to cancer evolution, adaptation and progression. Several lines of evidence indicate that immune cells play a protumor role in the development and metastasis of UM. UM cells may take advantage of immune privilege in the eye and escape immune surveillance even after leaving the niche [7].

Therefore, it is necessary to explore the interactive functions among the cell types and elucidate the molecular mechanism of the pathological changes in UM. In this study, we integrated TCGA data and four single-cell RNA sequencing (scRNA-seq) datasets containing one normal retinal pigment epithelium (RPE)/choroid dataset and three UM datasets to perform a detailed analysis of UM heterogeneity and intercellular communications that promoted cell state transitions. Our study may provide new signaling pathways and prognostic genes for UM treatment.

## Methods

### scRNA-seq data collection and quality control

The raw data in this study were downloaded from the GEO database (GSE138433, GSE139829, GSE160883, and GSE135133), which comprised a normal dataset containing 11 healthy retinal pigment epithelium (RPE)/choroid samples [8] and three UM datasets containing 20 primary UM samples and three metastatic UM samples [9–11]. We excluded low-quality cells based on the following criteria: (1) the number of features between 200 and the median ± 3 x median absolute deviation (MAD), (2) the counts and the percentage of mitochondrial and ribosomal genes were smaller than the median ± 3 x MAD, (3) all cells expressing hemoglobin genes were excluded, and (4) a sample in the GSE160883 database was dropped due to having too few cells. This resulted in 222,075 single cells being considered for further study (11 healthy samples from GSE135133, 6 primary UM samples from GSE138433, 5 primary UM samples from GSE160883, 8 primary UM samples and 3 metastatic UM samples from GSE139829). The data information was summarized in Table S1.

### Analysis of scRNA-seq data

The Seurat package (4.0.5) [12, 13] was used in R (version 4.1.1) for processing the data from the four datasets. The 33 samples were integrated by the Harmony R package [14] (0.1.0). After integration, the genes were summarized by principal component analysis (PCA) to reduce dimensionality. The first 30 principal components were used as input for cell clustering, and the cells were visualized in a two-dimensional uniform manifold approximation and projection (UMAP) representation. Cell types were annotated using canonical marker genes.

### TCGA data collection and processing

The human UM samples data were downloaded from The Cancer Genome Atlas (TCGA) database (https://portal.gdc.cancer.gov/). The “Alive, no UM metastasis” group (*n* = 50) and “Death, metastatic UM” group (*n* = 20) were obtained for further analysis. Other groups were dropped due to having too few samples. The log2(x + 1)-transformed gene expression of RNA-seq was restored to count data, and the DESeq2 package (1.32.0) was used for differentially expressed gene (DEG) analysis.

### Functional annotation analysis

To enable the functional analysis of DEGs, we used the R package clusterProfiler (4.0.5) [15, 16] to perform GO (Gene Ontology) enrichment analysis. The subontology Biological Process was specifically focused. A *P* value < 0.05 was considered statistically significant.

### Survival analysis

Kaplan–Meier survival analysis was performed for the UM patients. The Survminer package (0.4.9) was used for Kaplan–Meier analysis. The cutpoint was determined by the surv_cutpoint function.

### Cell–cell communication analysis

To identify and visualize cell–cell interactions between primary and metastatic UM samples, we employed the R package CellChat (1.1.3) [17]. We performed CellChat analysis according to the official workflow. All databases, including “Secreted Signaling”, “ECM-Receptor”, and “Cell–Cell Contact”, were used.

### Sample collection

This study was approved by the Ethical Committees of Yuncheng Central Hospital, Shanxi Medical University. Three primary UM tissue specimens were obtained from patients who underwent surgical resection at Yuncheng Central Hospital. The tumor tissues and adjacent normal tissues were separated by experienced doctors according to their gross appearance and stored immediately after the operation for cryopreservation. All samples were pathologically confirmed. The patient information was summarized in Table S2.

### Cell culture

MUM2B cells were purchased from iCell Bioscience (iCell-h148, iCell Bioscience, Shanghai, China) and cultured in 1640 medium containing 10% fetal bovine serum (FBS) and 1% penicillin/streptomycin (Gibco, Invitrogen, Carlsbad, CA, USA).

### siRNA transfection

CD44 and SPP1 siRNAs were synthesized by GenePharma (Shanghai, China) and transfected individually at 50 nM using Lipofectamine RNAiMAX transfection reagent (13778–100, Thermo Fisher Scientific) according to the manufacturer’s instructions. The siRNA sequences used in this study are listed in Table S3.

### Quantitative real-time polymerase chain reaction (qRT–PCR)

Total RNA was extracted using TRIzol (Invitrogen, Carlsbad, CA, USA) and reverse-transcribed by the PrimeScript RT reagent Kit (RR037A, TaKaRa, Kyoto, Japan). The quantification of target mRNA was performed by SYBR Green-based detection. The mRNA expression level was calculated using the 2 − (∆∆Ct) method. GAPDH was used as an internal reference gene. The primers used in this study are listed in Table S4.

### CCK-8 assay

Cell growth and viability were measured using a CCK-8 kit (Beyotime, Zhejiang, China) according to the manufacturer’s instructions. Briefly, 5 × 10^3^ MUM2B cells/well were seeded in a 96-well flat-bottomed plate and transfected with CD44 or SPP1 siRNAs for 24 h. Subsequently, 10 μL CCK-8 dye was added to each well and incubated for 1 h. Cell viability was determined by measuring the absorbance at 450 nm using a microplate reader.

### EdU proliferation assay

To assess cell proliferation, MUM2B cells were seeded in 24-well plates and transfected with CD44 or SPP1 siRNAs for 48 h. Then, cell proliferation was detected using the incorporation of 5-ethynyl-2′-deoxyuridine (EdU) with Click-iT EdU Assays (Invitrogen, Carlsbad, CA, USA) according to the manufacturer’s instructions. Briefly, the cells were incubated with EdU for 24 h, fixed in 4% paraformaldehyde, and permeabilized with 0.4% Triton in PBS. Then, the cells were incubated in 1× Click-iT Reaction Buffer and washed with PBS. The cell nuclei were stained with DAPI (Sigma). The proportion of EdU-labeled cells was determined by fluorescence microscopy.

### Cell migration assays

MUM2B cell migration was analyzed with a Transwell assay using a 24-well transwell with 8.0 μm pores (#3422, Corning Life Sciences, Corning, NY). MUM2B cells transfected with CD44 or SPP1 siRNAs were cultured for 24 h in RPMI 1640 supplemented with 10% FBS. Afterward, the cells were added to the top chamber with medium containing 2% FBS. Medium containing 10% FBS was added to the lower well. After culture for 12 h, the upper chamber was fixed with paraformaldehyde for 15 min and stained with 0.1% crystal violet. Cells that migrated to the bottom side of the membrane were counted in five random fields of view. The cell migration activity was described as the relative cell numbers of the transmitted cells.

### Statistical analysis

Statistical analysis of the data was performed using GraphPad Prism 8 (GraphPad Software, San Diego, CA) with an unpaired t test or one-way ANOVA. *P* values lower than 0.05 were considered significant.

## Results

### Integration of multiple single-cell transcriptomic data revealed the complexity of UM

**Fig. 1:**
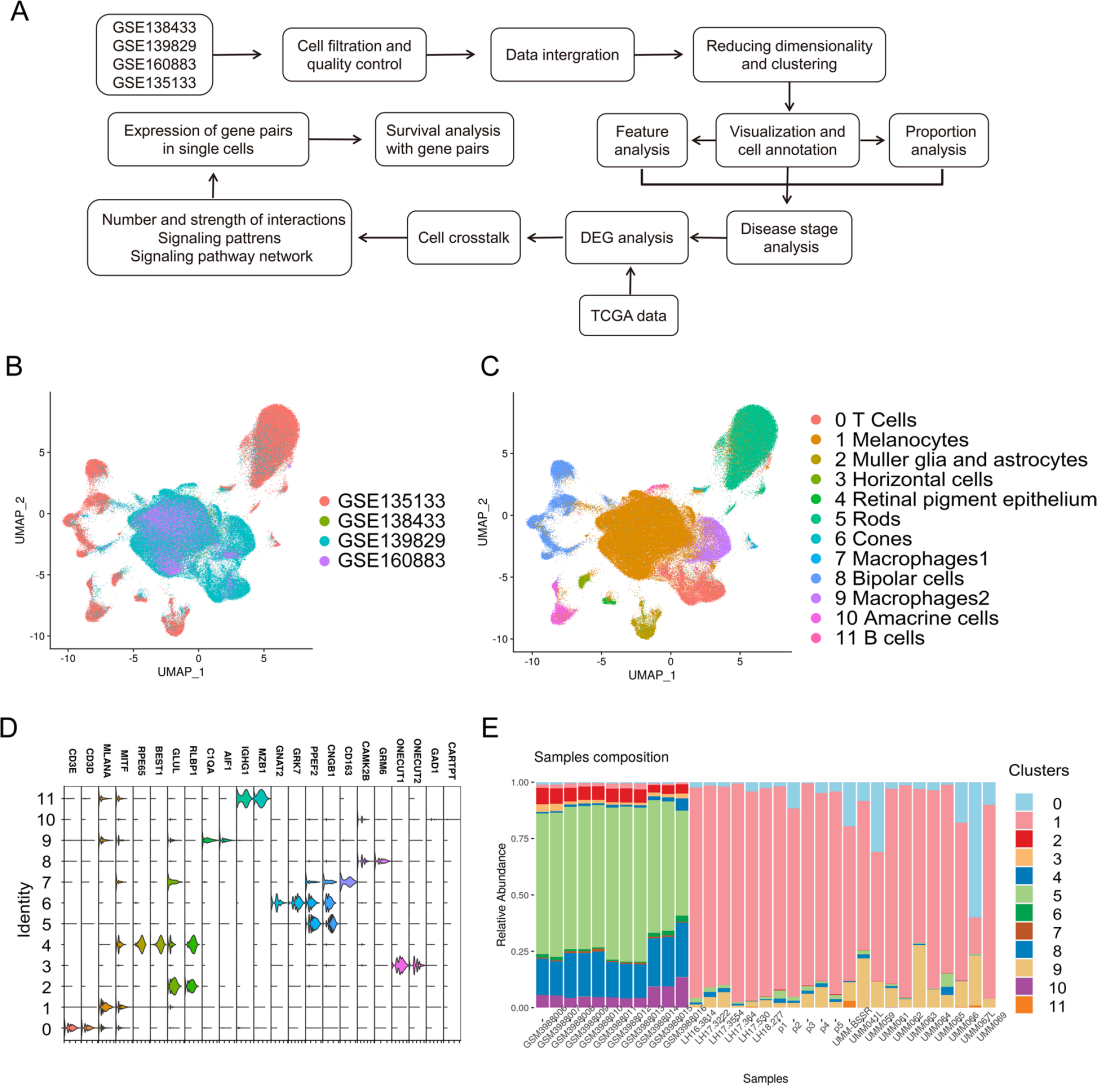
The integration and analysis of the scRNA-seq data from 33 samples in four datasets. **A** Workflow of single-cell data processing and analysis. **B** UMAP plot showing the integration of four datasets. **C** UMAP visualization of 222,075 cells, colored by cluster identity, and annotated on the basis of marker genes (**D**). **E** The composition of each cluster in each sample

UM progression and treatment failure are often related to the heterogeneity of tumor cells [18]. We downloaded scRNA-seq data from 33 samples (11 healthy samples and 22 UM patients) in four datasets to delineate the cellular heterogeneity in retinal pigment epithelium (RPE)/choroid tissues. After stringent quality control, a total of 222,075 cells from 33 samples were retained for further analysis (Fig. 1 A and Fig. S1). To exclude the batch effects across the datasets, we performed integration analysis for the 33 samples with the sctransform method. As shown in Fig. 1 B, the four datasets were well integrated for all 33 samples (Fig. S2). The differentially expressed genes (DEGs) among cell clusters and highly specific marker genes were selected to annotate cell types. Some small clusters were merged according to the common specific marker genes. As a result, all cells were identified as 12 clusters (Fig. 1 C), and each cluster had a unique gene expression (Fig. 1 D). The vision-associated cell types, such as rods, cones, biopolar cells and glia, originated from the healthy data, whereas the UM datasets were mainly composed of melanocytes, T cells, B cells and macrophages (Fig. 1 E). These findings indicate the proliferation of melanocytes and the infiltration of immune cells in UM.

### Tumor stage-specific alteration in the cell ratio of UM

**Fig. 2:**
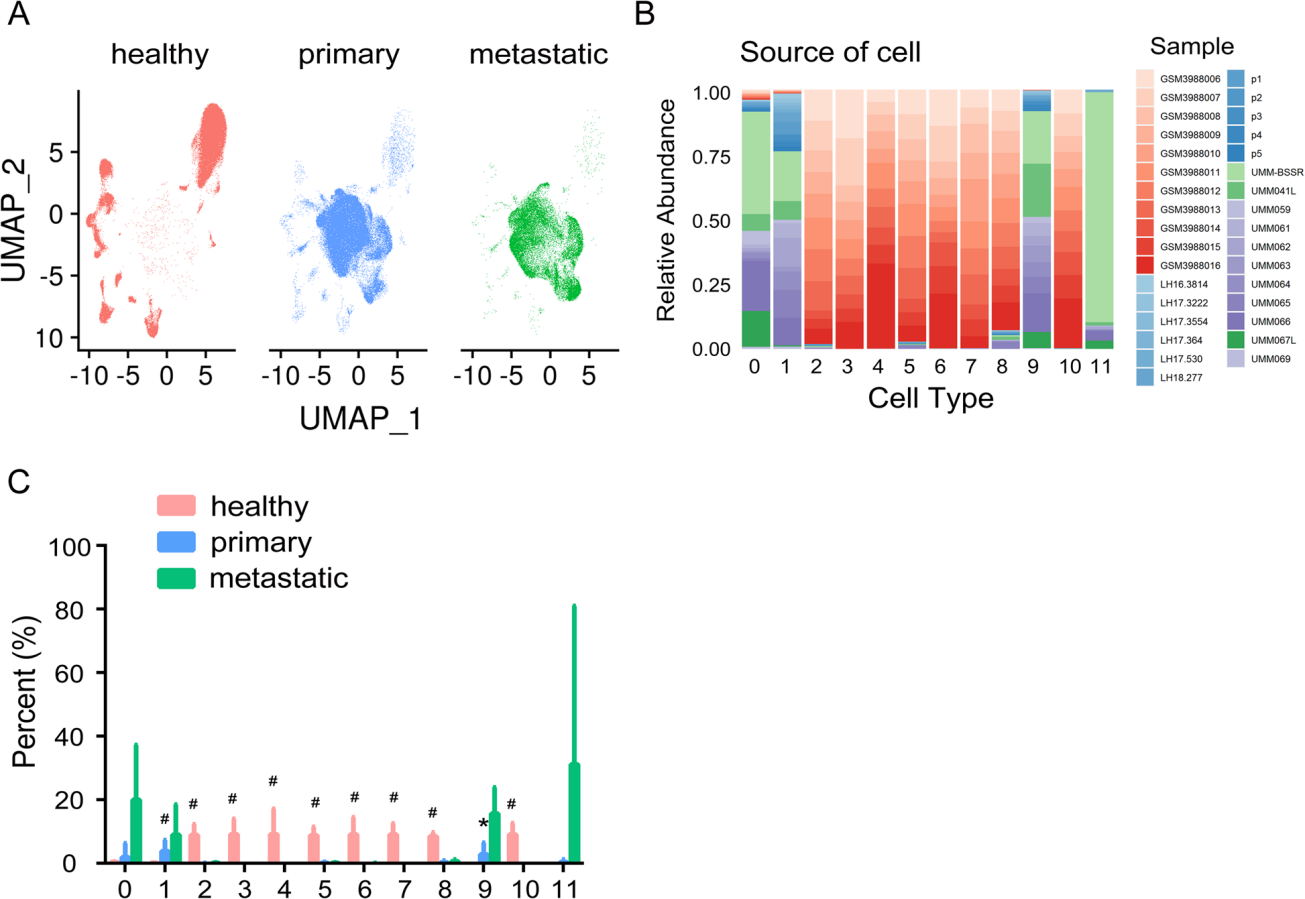
Cell composition in different tumor stages of UM. **A** UMAP plot showing the cell clustering in healthy samples and UM samples. **B** The composition of each sample in each cell cluster. **C** The percentage of healthy, primary and metastatic UM cells in each cluster. *, *P <* 0.05; #, *P <* 0.01

Most UM patients develop metastatic disease with survival prognoses of no more than one year [19], so we paid close attention to metastasis-related cell clusters. As the disease developed and progressively worsened, most vision-related cells in the normal eye structure disappeared and were replaced by melanocytes (Fig. 2 A). A large proportion of primary and metastatic UM samples were observed in immune-associated clusters (Fig. 2 B). We collected patient information and metadata and calculated the cell proportions of healthy, primary and metastatic UM samples in each cluster. Our data showed that while a significant reduction in vision-related clusters was demonstrated, such as in Cluster 2, Cluster 3, Cluster 4, Cluster 5, Cluster 6, Cluster 7, Cluster 8, and Cluster 10, the proportion of melanocytes in Cluster 1 and macrophages in Cluster 9 was remarkably elevated (Fig. 2 C). Of note, the proportion of T cells (Cluster 0) and B cells (Cluster 11) was not statistically significant in our data due to the large variance differences, but it strongly implied the infiltration of these immune cells.

### Identification of DEGs in each cell cluster and TCGA associated with a high risk of metastasis

**Fig. 3:**
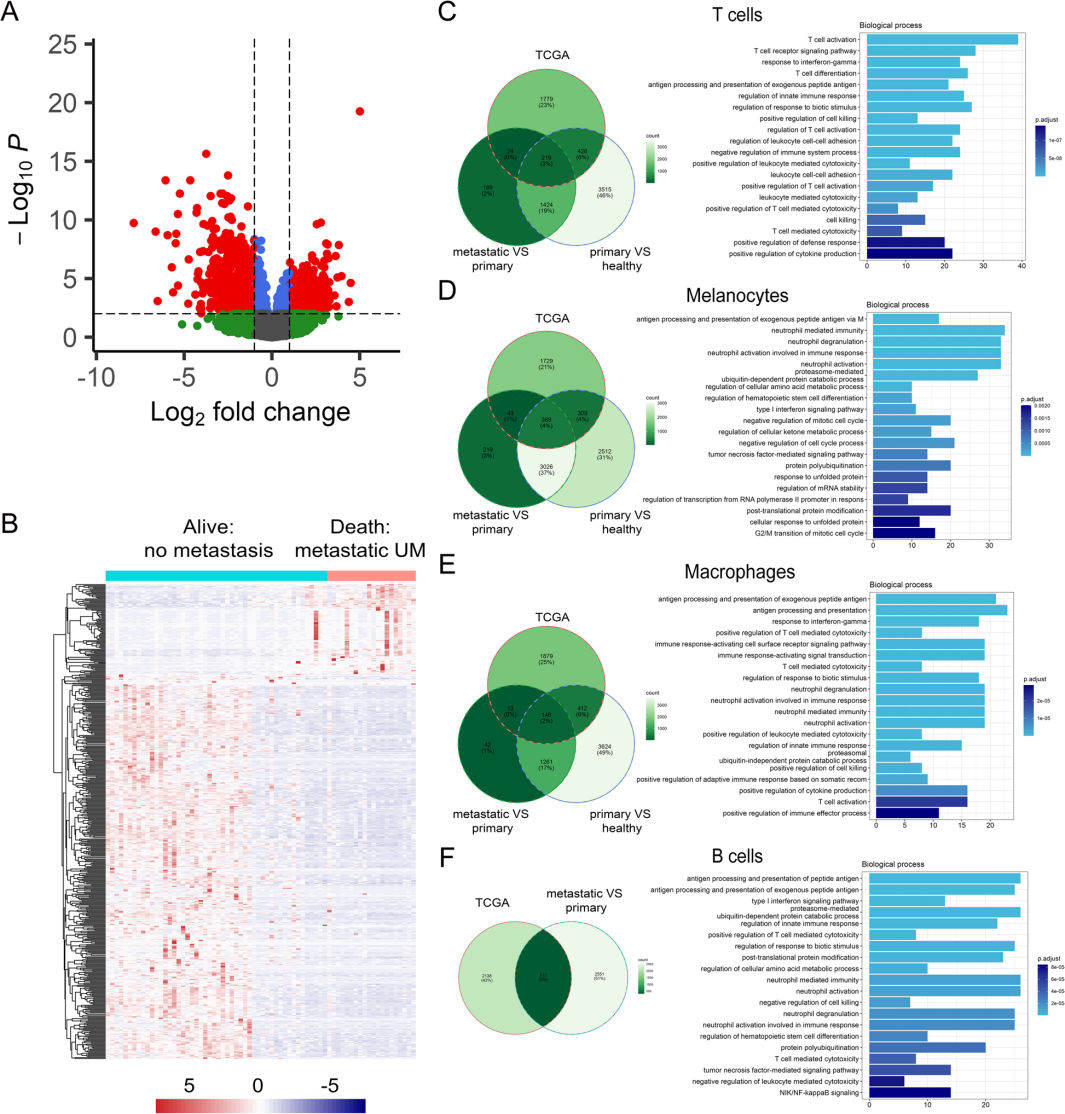
DEGs in TCGA data and scRNA-seq datasets. **A** Volcano plot of the DEGs in the “Alive, no UM metastasis” and “Death, metastatic UM” groups in TCGA data. **B** Heatmap depicting the top 500 most significant DEGs in the “Alive, no UM metastasis” group and the “Death, metastatic UM” group. **C**-**F** *Left*, Venn diagrams of the shared and unique DEGs among the indicated groups in each cell type; *right*, the functional annotations for genes in all three groups in each cell type

To reveal the DEGs in the UM tissues, we introduced the TCGA data. Gene expression was analyzed in the “Alive, no UM metastasis” (*n* = 50) and “Death, metastatic UM” (*n* = 20) groups. A total of 1328 genes were found to be differentially expressed (Table S5). As shown in Fig. 3 A, the volcano plot showed the upregulated and downregulated DEGs in the TCGA dataset with the cutoff criteria of *P <* 0.01 and |log2FC| > 1. Hierarchical clustering of the top 500 most significant DEGs indicated a greater similarity within each group and a significant difference between the non-UM metastasis and metastatic UM samples (Fig. 3 B).

We then analyzed the DEGs between primary and healthy samples and between metastatic and primary samples in each cluster (Table S6). Venn diagrams were used to demonstrate the overlapping DEGs among the three datasets, including significantly upregulated and downregulated genes. These DEGs were further applied to gene set enrichment in each cluster (Fig. 3 C-F). The results revealed the related molecular mechanisms and pathways of UM with distinct functional patterns. For example, the DEGs in the T cell cluster were significantly associated with T cell activation and the T cell receptor signaling pathway, and the DEGs in the B cell cluster were significantly correlated with antigen processing and presentation of peptide antigens. Interestingly, the melanocyte cluster is involved in the antigen processing and presentation of exogenous peptide antigen, suggesting reciprocal communication between melanocytes and immune cells.

### Identification of cell–cell communication between primary and metastatic UM

**Fig. 4:**
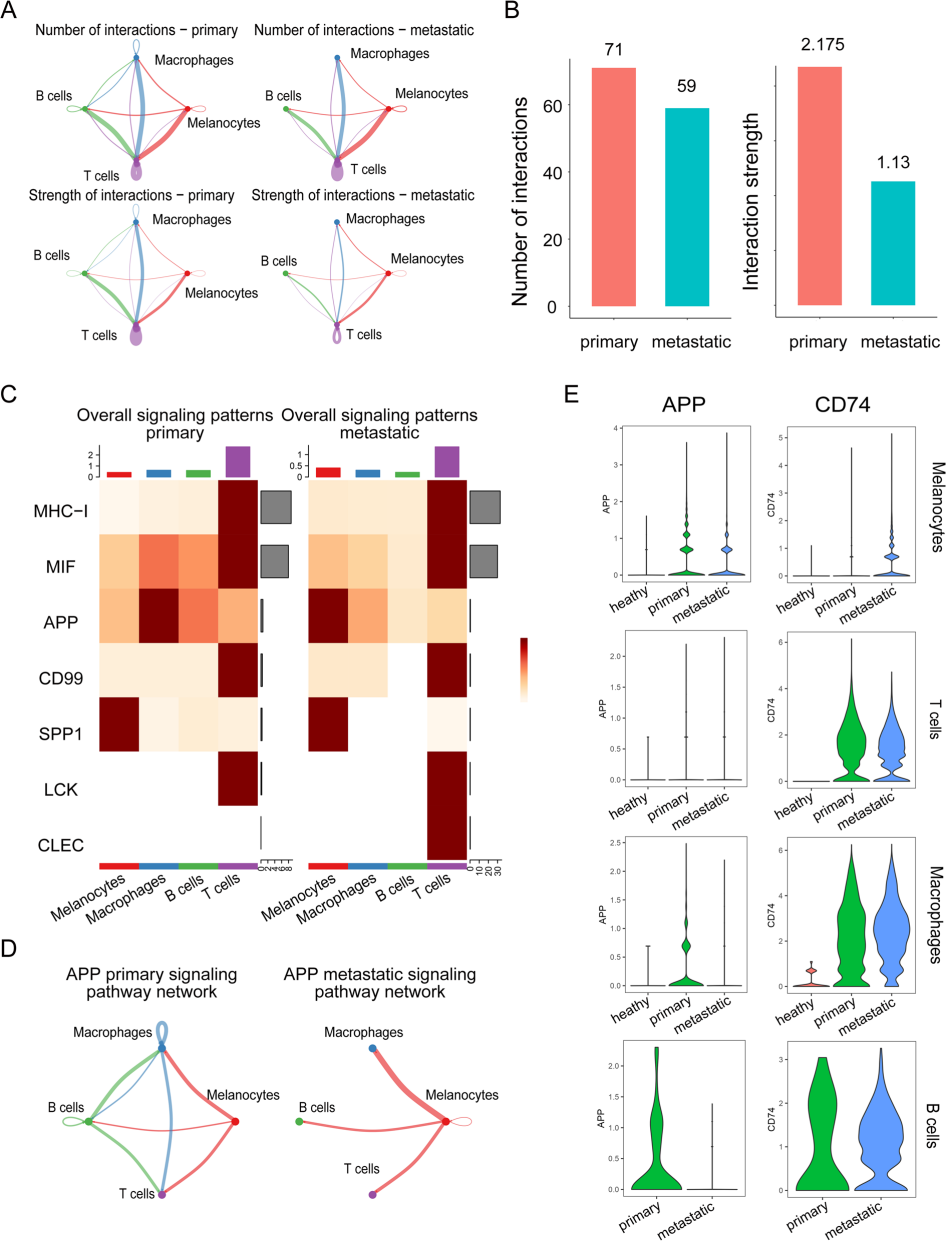
Cell–cell communication between primary and metastatic UM. **A** Diagrams displaying the interaction number and strength in cell clusters. **B** Bar plot showing the interaction number and strength between primary and metastatic UM. **C** Heatmap depicting signals contributing the most to the overall signaling pathways in primary and metastatic UM. **D** APP signaling in cell–cell interactions between primary and metastatic UM. **E** The expression of the gene pair APP and CD74 in melanocytes, T cells, macrophages, and B cells

Next, we aimed to identify alterations in cell–cell communication between primary and metastatic UM patients. The communication network between primary UM individuals and metastatic UM patients was constructed to characterize the alterations in signaling pathways (Fig. 4 A). The global number and strength of interactions were both reduced. Strikingly, the interaction strength in metastatic samples remained half of that in primary samples (Fig. 4 B). This was more obvious in the overall signaling patterns (Fig. 4 C). The alterations of signaling pathways were illustrated in the incoming and outgoing signaling patterns (Fig. S3). In particular, the MHC-I, MIF, APP, CD99, SPP1, LCK, and CLEC signaling pathways were actively involved in the outgoing and incoming signaling patterns between primary and metastatic UM patients. We further characterized each signaling pathway network and visualized the pathway genes in each cluster. As shown in Fig. 4 D, the cell interactions among T cells, B cells and macrophages were lost in metastatic UM samples compared to primary UM samples (Fig. 4 D). The expression of APP pathway genes, the APP ligand, and the receptor CD74 is shown in Fig. 4 E, revealing that the destruction of the APP pathway was due to the reduction in APP in macrophages and B cells. In contrast, CD74 expression was increased significantly. The analysis of the signaling pathway network and pathway genes in single cells showed the general destruction of the immune network (Fig. S4).

### Validation of ligand–receptor pairs in UM patients

**Fig. 5:**
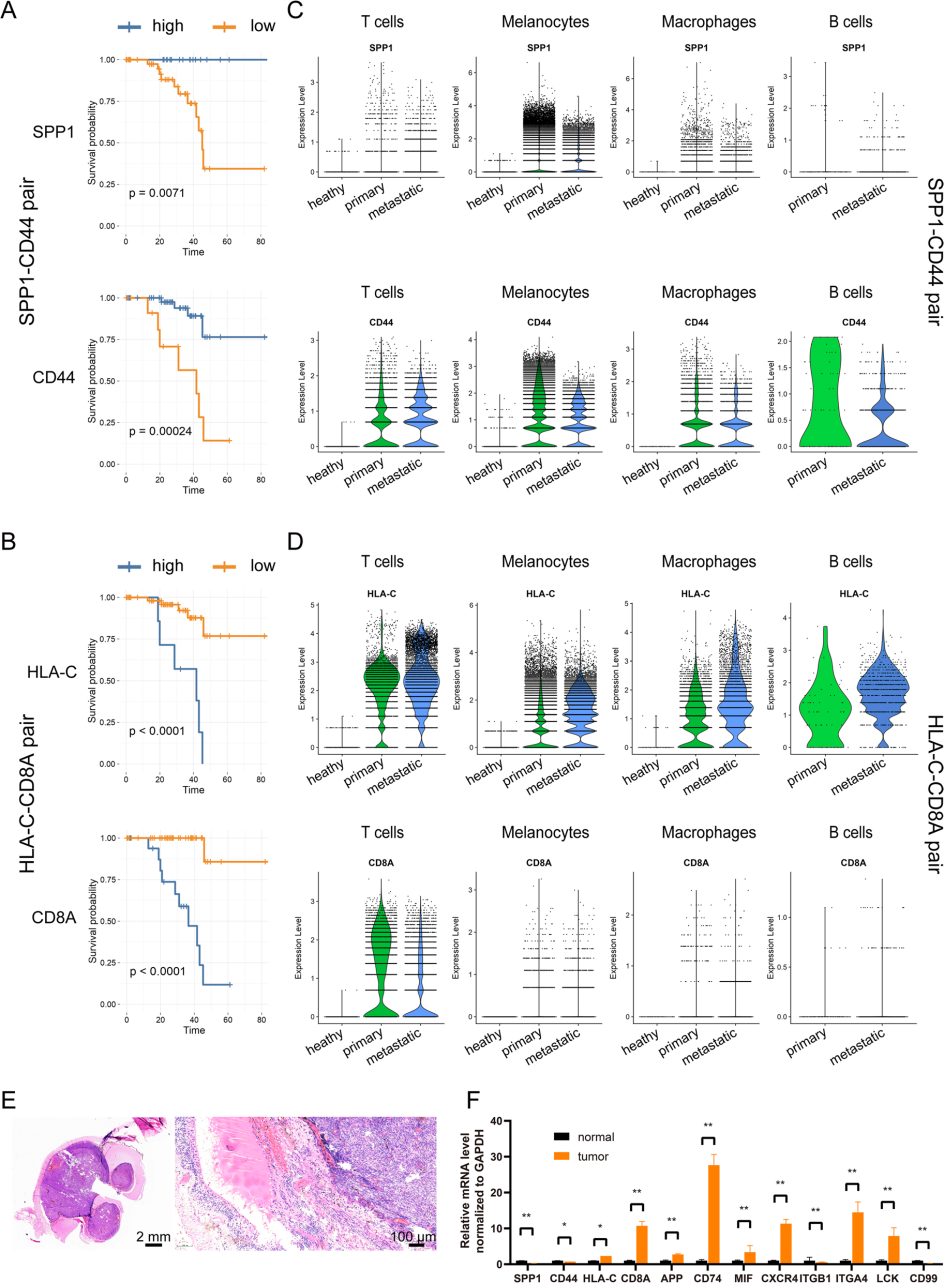
Survival analysis for key signaling pathways in UM patients. Kaplan–Meier survival analysis of the SPP1-CD44 gene pair (**A**) and HLA-C-CD8A gene pair (**B**) was performed to estimate the OS of high-risk and low-risk patients in the TCGA cohort. Expression of the SPP1-CD44 gene pair (**C**) and HLA-C-CD8A gene pair (**D**) in melanocytes, T cells, macrophages, and B cells. (**E**) H&E staining for the UM samples. (**F**) qPCR detection of gene pairs in the signaling pathway in UM and adjacent tissues

To determine the correlation between the ligand–receptor pairs in signaling pathways and the clinical characteristics of UM patients, Kaplan–Meier survival analysis was conducted. The clinical information of 80 UM patients was collected from the TCGA database for the overall survival analysis. The results confirmed that the mRNA expression of these gene pairs was tightly correlated with the survival rate of UM (Fig. 5 A-B and Fig. S5). The decreased expression of the SPP1 signaling pathway, including the ligand SPP1 and the receptor CD44, was closely associated with poor overall survival in UM (Fig. 5 A), whereas the decreased expression of the MHC-I pathway, including HLA-C and CD8A, predicted a better survival rate (Fig. 5 B). We further investigated the contribution of each cell cluster to the overall elevation of SPP1 and CD44. The single-cell analysis showed that the attenuation of the SPP1 signaling pathway originated from the decrease in the expression of the pathway gene in melanocytes (Fig. 5 C). Interestingly, while the mRNA level of HLA-C was downregulated in melanocytes, its expression in immune cells was upregulated (Fig. 5 D), suggesting dysfunction of the immune response. The signaling gene pairs were also validated in UM samples. We obtained UM tissues (Fig. 5 E) and checked the gene expression of signaling gene pairs, such as SPP1-CD44, HLA-C-CD8A, in tumor and adjacent normal tissues. Consistent with the results observed in the sequencing data, these signaling genes were significantly altered (Fig. 5 F). Taken together, our results suggest that the signaling pathways are abnormally altered in UM samples.

### SPP1 signaling pathway in melanocytes

**Fig. 6:**
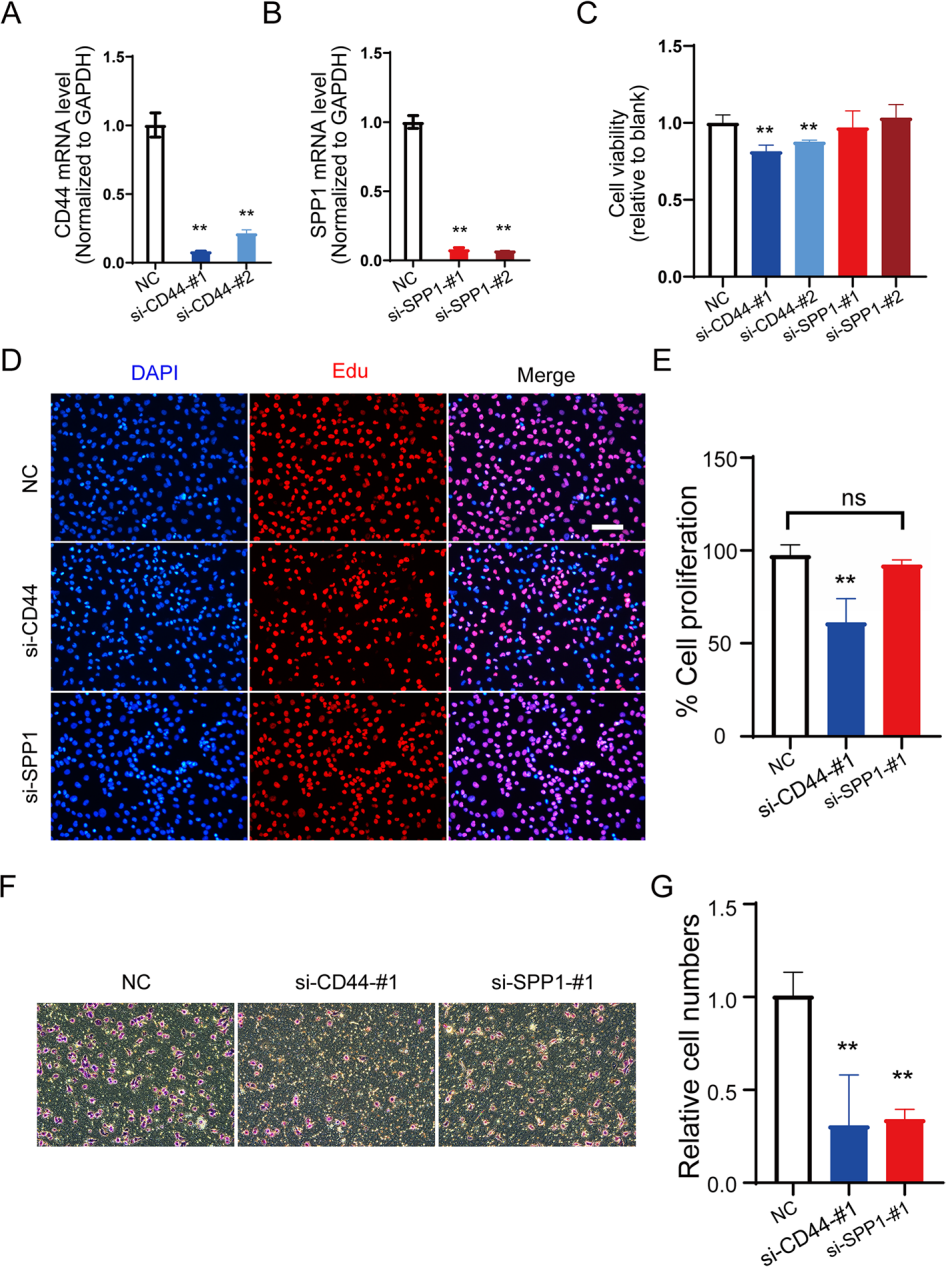
The function of the SPP1 signaling pathway in melanocytes. Knockdown efficiency of CD44 (**A**) and SPP1 (**B**) in melanocytes. **C** Cell viability was determined 24 h after CD44 and SPP1 knockdown, followed by a CCK-8 assay. Data were normalized to NC. **D** Immunofluorescence staining using EdU in CD44- and SPP1-silenced melanocytes. Scale bars: 100 μm. **E** Data were collected from at least five sections, and cell numbers were averaged. **F** The migration activity of melanocytes transfected with CD44 siRNA or SPP1 siRNA was measured by a Transwell assay. **G** Data are shown as the mean ± SD of three independent experiments. **, *P <* 0.01

To reveal the function of SPP1 signaling in melanocytes, we silenced the expression of SPP1 signaling genes, ligand CD44 and receptor SPP1, to measure the proliferation and migration in melanocytes. The knockdown efficiency was evaluated with quantitative PCR (qPCR), and the gene expression of CD44 and SPP1 was dramatically decreased by siRNA application (Fig. 6 A, B). Next, we examined the effects of SPP1 pathway interference on melanocyte proliferation. As shown in Fig. 6 C, both of the siRNA sequences targeting CD44 significantly lowered the viability of melanocytes, whereas the knockdown of SPP1 did not significantly affect the proliferation of MUM2B cells, as detected by the CCK-8 assay. In line with the results of the CCK-8 assay, MUM2B cells transfected with CD44 siRNA showed a remarkable decrease in EdU incorporation, but melanocytes with SPP1 ablation demonstrated a growth rate comparable to that of the control cells (Fig. 6DE). We further explored the influence of SPP1 signaling on melanocyte migration. The Transwell assay illustrated that knockdown of CD44 or SPP1 notably prevented cancer cell migration (Fig. 6FG). These results indicate that the SPP1 pathway plays a pivotal role in proliferation and migration in UM and that inhibition of this signaling pathway may favor the treatment of UM.

## Discussion

UM is a malignant tumor with high mortality and ocular morbidity [20]. The easy invasion and metastasis of UM seriously threatens the vision and life of patients [21]. Therefore, accurate prognostic biomarkers and early diagnosis are of great significance for the reduction of distant metastasis and promotion of UM treatment. Although great progress has been made for local control of primary disease, the survival rate after a diagnosis of metastatic UM is not satisfactory. The mechanisms of action, regulation and metastasis need to be elucidated to determine new diagnosis and treatment options.

The present study provides several lines of evidence for understanding the pathogenesis of UM. First, we reveal the abnormal signaling pathway in UM samples. Using CellChat, we inferred intercellular communications and compared the healthy and diseased samples in UM to provide a new vision for recognizing the mechanism of UM. Second, we combined the bulk-seq analysis of UM with the scRNA-seq data, depicting the heterogeneity of the tumor tissues. Third, we attribute the contribution of the differentially expressed genes to each cell type and analyze the function of these genes in specific cell types. These findings suggest that the dysfunction of cell-cell communication, especially the inability or responsiveness of melanocytes and immune cells, may contribute to the exacerbation of UM.

In this study, we took advantage of scRNA-seq technology to integrate four high-throughput transcriptome datasets to reveal the molecular features in healthy RPE/choroid tissue and primary and metastatic UM tissues. We included 222,075 single cells obtained by dynamic filtration from 33 samples (11 healthy, 19 primary and 3 metastatic samples). These cells were classified into 12 clusters (vision-associated cells and immune-associated cells), which indicated high heterogeneity in the progression of UM. Heterogeneity is one of the hallmarks of malignant tumors, which can cause tumor growth, invasion, and metastasis. Melanocyte carcinogenesis is paralleled by immune cell infiltration. As normal vision-related cells, such as cones and rods, diminished, the melanocytes expanded, accompanied by an increase in B cells, T cells and macrophages.

We introduced bulk RNA-seq data from TCGA. The DEGs were obtained by comparing the “alive: no metastasis” and “death: metastasis” groups. These DEGs were further validated in single-cell data in melanocytes and immune cell clusters to reveal their functions. Our GO analysis demonstrated that these cells were involved in a series of immune processes: the T cell receptor signaling pathway, T cell differentiation, antigen processing and presentation, neutrophil activation in melanocytes, antigen processing and presentation in macrophages, and the type I interferon signaling pathway in B cells. These data implied that the immune response could play a key role in the development of UM. However, evidence has shown limited clinical efficacy with no benefit to OS for metastatic UM. Low response rates of checkpoint inhibition in advanced UM are observed in clinical practice.

To uncover the mechanism of the low immune response in UM, we constructed a cell crosstalk network by CellChat. Our results found that the overall number and strength of the interaction in metastatic UM were significantly attenuated compared to those in primary UM. Various signaling pathways, such as MHC-I, MIF, APP, CD99, SPP1, LCK, and CLEC, were impaired, especially the communication between melanocytes and immune cells. Previous studies have demonstrated the presence of immune infiltrates in primary and metastatic UM, but immune checkpoint blockade failed to achieve impressive OS in UM. To further explore the possible molecular mechanisms of nonresponsiveness in these immune cells, we analyzed the expression of signaling pathway genes at the single-cell level. As expected, substantial alterations in mRNA expression were observed in immune cells and melanocytes, which corresponded to the survival rate of UM patients.

For instance, the overall high expression of HLA-C and CD8A leads to a shorter OS. The high expression of HLA-C is attributed to T cells, B cells and macrophages but not melanocytes, suggesting immune dysfunction in the microenvironment. The SPP1-CD44 gene pair is another example. The low expression of both genes in melanocytes may be responsible for the poor OS, which indicates inadequate immune stimulation.

SPP1 (secreted phosphoprotein 1), also called osteopontin, is a member of the small integrin-binding ligand, N-linked glycoprotein family of proteins [22]. The gene is located on chromosome 4 (4q21-4q25) in humans and has five alternatively spliced transcripts. As a multifunctional phosphoglycoprotein, it is expressed in a wide range of cells, including osteoblasts, neurons, epithelial cells, and lymphoid cells. The SPP1 gene has been reported to participate in cancer development and progression [23]. It is often overexpressed in the tumor microenvironment and elevated in the peripheral blood. Overexpression of SPP1 is linked to worse prognosis in many cancers, including hepatocellular carcinoma, colorectal cancer, lung cancer, breast cancer, bladder cancer, and acute myeloid leukemia [22, 24]. CD44 is a nonkinase transmembrane glycoprotein encoded by the *CD44* gene located on the short arm of chromosome 11p13 [25]. CD44 was first identified on lymphocytes and is overexpressed in several cell types, including cancer stem cells [26]. It is recognized as a cell adhesion protein and is involved in cell–cell and cell-matrix interactions [27]. Our results showed that SPP1-CD44 signaling, at least in part, contributed to the malignancy of UM through the promotion of proliferation and metastasis. Therefore, understanding the function and regulation of the SPP1 pathway in UM will provide potential treatment options in the future.

One of the striking features of UM is the infiltration of inflammatory and immune cells that promote tumor progression [28–30]. Our data confirmed this result. The average proportions of lymphoid cells and myeloid cells were 0.35 and 9.08% in healthy samples, respectively. Interestingly, lymphoid cells increased to 2.23%, whereas myeloid cells decreased to 2.80% in primary UM samples. Both cell types were elevated dramatically in metastatic UM samples (more than 7% in lymphoid cells and 15% in myeloid cells). Although active infiltration of inflammatory and immune cells occurs in tumor tissues, the prognosis of metastatic UM is not favorable because melanocytes in UM have relatively few tumor-specific neoantigens that can be presented to the immune system and therefore they evade immune attack [31, 32]. This was observed in our data, where we showed that the CD8A-HLA-C gene pair was significantly increased in T/B cells but decreased in melanocytes, indicating the impairment of normal signaling transduction.

The genetic background contributes to the progression of UM. The most commonly mutated genes in UM are GNA11, GNAQ, BAP1, EIF1AX, and SF3B1. Evidence has shown that BAP1 mutation or loss is linked to metastatic UM and associated with poor prognoses [6, 33]. Immune genes were significantly correlated with both BAP1 expression and chromosome 3 copy number variation [34]. However, due to the paucity of mutation information in scRNA-seq information, we could not evaluate the genetic background across the datasets. Moreover, as a 3′ mRNA-seq, the 10X Genomics single-cell sequencing does not have a great change to detect SNP. Smart-seq may be more suitable for mutation detection. The original paper performed copy number variation analysis of primary and metastatic UM samples, so we did not repeat the analysis. In this revision, we supplemented the gene expression of the commonly mutated genes (Fig.S6), indicating a differential regulation of these gene in various cell types.

## Conclusions

In conclusion, we performed a comprehensive analysis of the scRNA-seq datasets of UM, illustrated the cellular heterogeneity of UM tissues, and revealed dysfunction of the immune signaling pathway in UM. Different mechanisms may exist in the disturbance of immune balance and the impairment of the immune system. A series of gene pairs in signaling pathways could serve as potential novel biomarkers for risk assessment and diagnosis. These pathways might constitute an additional therapeutic strategy to the current treatment of UM.

## Supplementary Information

**Additional file 1: Table_S1.** Summary information for the 4 original scRNA-seq datasets and integrated dataset

**Additional file 2: Table_S2.** Clinical information and gene-expression profile of the three primary uveal melanomas

**Additional file 3: Table_S3.** Sequences of siRNA used in this study

**Additional file 4: Table_S4.** Primers for real-time PCR

**Additional file 5: Table_S5.** Differentially expressed genes in TCGA data_UVM

**Additional file 6: Table_S6.** Differentially expressed genes in each cluster

**Additional file 7: Fig. S1.** The quality control of integrated datasets. The four datasets containing 11 healthy and 22 UM samples were integrated and filtered according to strict dynamic filtration. **Fig. S2.** The cell distribution of each sample in integrated UMAP. The UMAP plot showing the cell distribution of each sample. **Fig. S3.** The incoming and outgoing signaling patterns. The heatmap depicting the incoming and outgoing signaling patterns in primary and metastatic UM. **Fig. S4.** The signaling pathway network. The aberrant signaling pathways including (A) CD99, (B) MIF, (C) LCK, (D) MHC-I, and (E) SPP1. **Fig. S5.** The survival curves of signaling pathway genes. Kaplan-Meier survival analysis of the signaling pathway associated genes. **Fig. S6.** The gene expression of most commonly mutated genes in UM. Violin plot showing the gene expression of GNA11, GNAQ, BAP1, EIF1AX, and SF3B1 in normal, primary and metastatic tissue of UM

## Data Availability

The single-cell RNA sequencing data in this study were available from the public database GEO (https://www.ncbi.nlm.nih.gov/geo/), and the TCGA data were obtained from UCSC XENA website (https://xenabrowser.net/datapages/).
