# Supplementary material for: Integrated analysis reveals the dysfunction of signaling pathways in uveal melanoma
Source: BMC Cancer. 2022 Jul 5;22:734. doi: 10.1186/s12885-022-09822-8 (PMC9258069; doi:10.1186/s12885-022-09822-8)
Supplement: Supplementary file 3 — Additional file 3: Table_S3. Sequences of siRNA used in this study [file 12885_2022_9822_MOESM3_ESM.docx]

Table S3. Sequences of siRNA used in this study

| Gene name | Gene ID | siRNA |
| --- | --- | --- |
| si-CD44-#1 | 960 | 5’-TATTCCACGTGGAGAAAAA-3’ |
| si-CD44-#2 | 960 | 5’-GAAACTCCAGACCAGTTTA-3’ |
| si-SPP1-#1 | 6696 | 5’-GCTACAGACGAGGACATCA-3’ |
| si-SPP1-#2 | 6696 | 5’-CCGTGGGAAGGACAGTTAT-3’ |
