# Supplementary material for: Integrated analysis reveals the dysfunction of signaling pathways in uveal melanoma
Source: BMC Cancer. 2022 Jul 5;22:734. doi: 10.1186/s12885-022-09822-8 (PMC9258069; doi:10.1186/s12885-022-09822-8)
Supplement: Supplementary file 4 — Additional file 4: Table_S4. Primers for real-time PCR [file 12885_2022_9822_MOESM4_ESM.docx]

Table S4. Primers for real-time PCR

| Gene name | Forward Primer (5' to 3' ) | Reverse Primer (5' to 3' ) |
| --- | --- | --- |
| CD44 | 5′-AAAGGAGCAGCACTTCAGGA-3′ | 5′-TGTGTCTTGGTCTCTGGTAGC-3′ |
| SPP1 | 5′-CGAGGTGATAGTGTGGTTTATGG-3′ | 5′-GCACCATTCAACTCCTCGCTTTC-3′ |
| HLA-C | 5′-AGATCACCCAGCGCAAGTT-3′ | 5′-CGTCTCCTTCCCGTTCTCC-3′ |
| CD8A | 5′-GCTGGATCGGACCTGGAA-3′ | 5′-AGGAGGAAGGTGGGACTGG-3′ |
| APP | 5′-TTTGTGATTCCCTACCGC-3′ | 5′-GTGCCAGTGAAGATGAGTTT-3′ |
| CD74 | 5′-GCACCATTGGCTCCTGTT-3′ | 5′-TGTGGCTGACCTCTTCCTG-3′ |
| MIF | 5′-CACAGCATCGGCAAGATCG-3′ | 5′-GGAGTTGTTCCAGCCCACATT-3′ |
| CXCR4 | 5′-CCCCATCCTCTATGCTTT-3′ | 5′-GAATGTCCACCTCGCTTT-3′ |
| ITGB1 | 5′-GCACGATGTGATGATTTA-3′ | 5′-CTTTGCTACGGTTGGTTA-3′ |
| ITGA4 | 5′-AGCCCTAATGGAGAACCT-3′ | 5′-TGCCCACAAGTCACGATG-3′ |
| LCK | 5′-CTGGTTCGGCTCTACGCT-3′ | 5′-CCATGTCCAGGAGTTTGTTG-3′ |
| CD99 | 5′-GGGATGACTTTGACTTAGGAG-3′ | 5′-GAGGAACTAGGGTGGTTGG-3′ |
| GAPDH | 5′-GCGAGATCCCTCCAAAATCA-3′ | 5′-ATGGTTCACACCCATGACGA-3′ |
