# Supplementary material for: Integrated analysis reveals the dysfunction of signaling pathways in uveal melanoma
Source: BMC Cancer. 2022 Jul 5;22:734. doi: 10.1186/s12885-022-09822-8 (PMC9258069; doi:10.1186/s12885-022-09822-8)
Supplement: Supplementary file 7 — Additional file 7: Fig. S1. The quality control of integrated datasets. The four datasets containing 11 healthy and 22 UM samples were integrated and filtered according to strict dynamic filtration. Fig. S2. The cell distribution of each sample in integrated UMAP. The UMAP plot showing the cell distribution of each sample. Fig. S3. The incoming and outgoing signaling patterns. The heatmap depicting the incoming and outgoing signaling patterns in primary and metastatic UM. Fig. S4. The signaling pathway network. The aberrant signaling pathways including (A) CD99, (B) MIF, (C) LCK, (D) MHC-I, and (E) SPP1. Fig. S5. The survival curves of signaling pathway genes. Kaplan-Meier survival analysis of the signaling pathway associated genes. Fig. S6. The gene expression of most commonly mutated genes in UM. Violin plot showing the gene expression of GNA11, GNAQ, BAP1, EIF1AX, and SF3B1 in normal, primary and metastatic tissue of UM [file 12885_2022_9822_MOESM7_ESM.pdf]

FigureS1

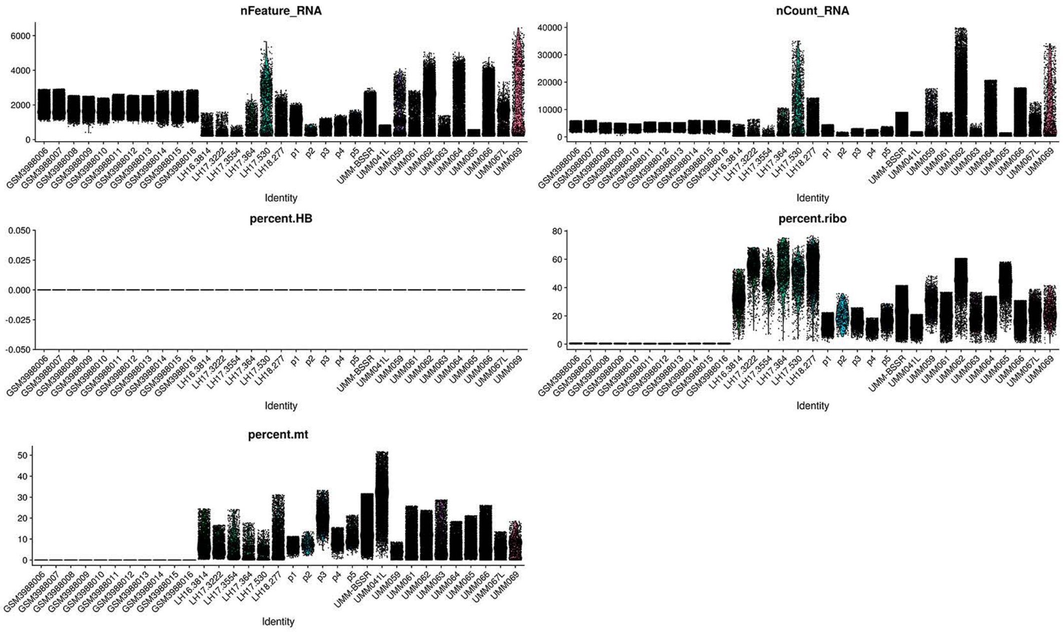

nFeature_RNA
nCount_RNA
percent.HB
percent.ribo
percent.mt
Identity

FigureS2

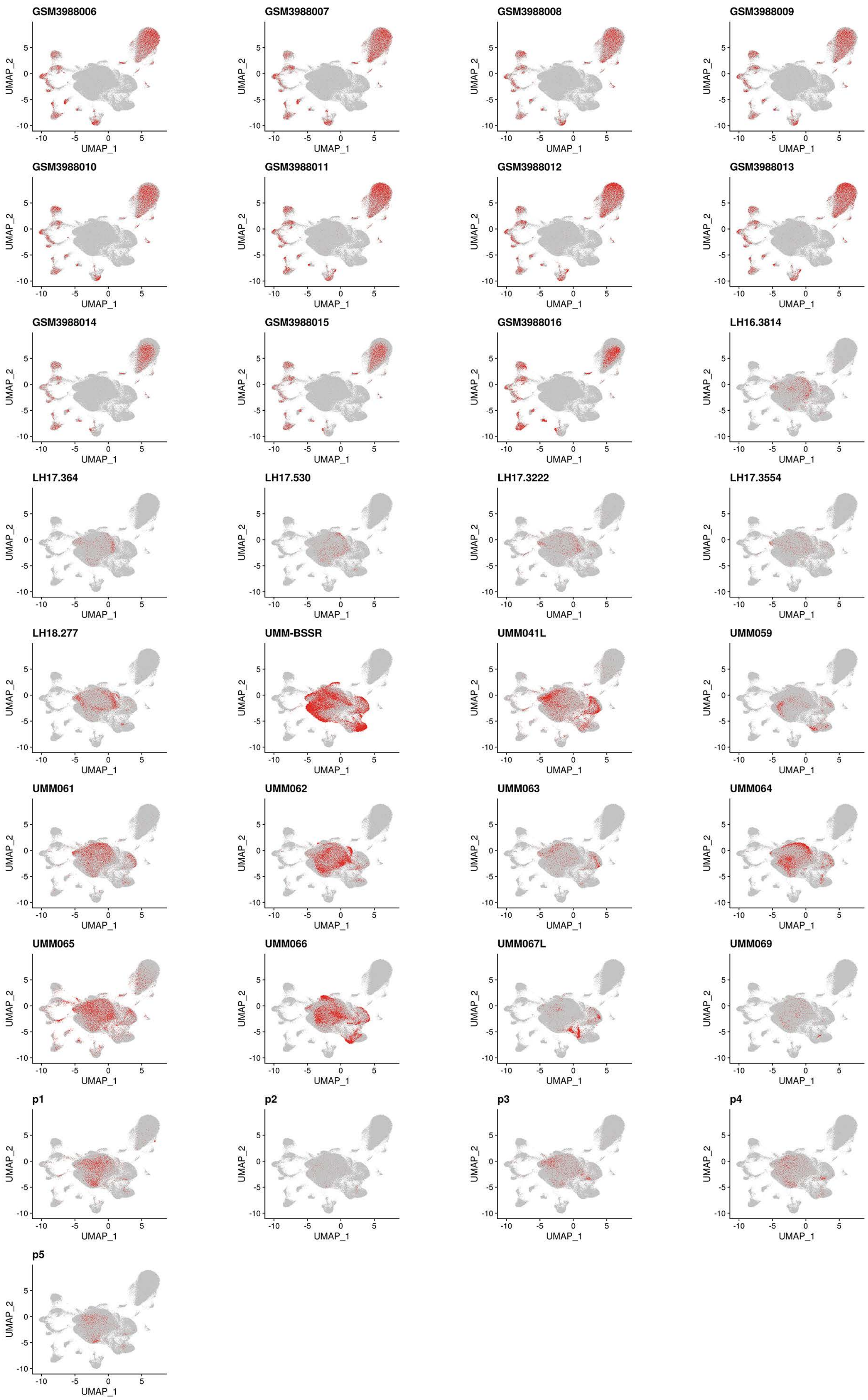
GSM3988006
GSM3988007
GSM3988008
GSM3988009
GSM3988010
GSM3988011
GSM3988012
GSM3988013
GSM3988014
GSM3988015
GSM3988016
LH16.3814
LH17.364
LH17.530
LH17.3222
LH17.3554
LH18.277
UMM-BSSR
UMM041L
UMM059
UMM061
UMM062
UMM063
UMM064
UMM065
UMM066
UMM067L
UMM069
p1
p2
p3
p4
p5
UMAP_1
UMAP_2

FigureS3

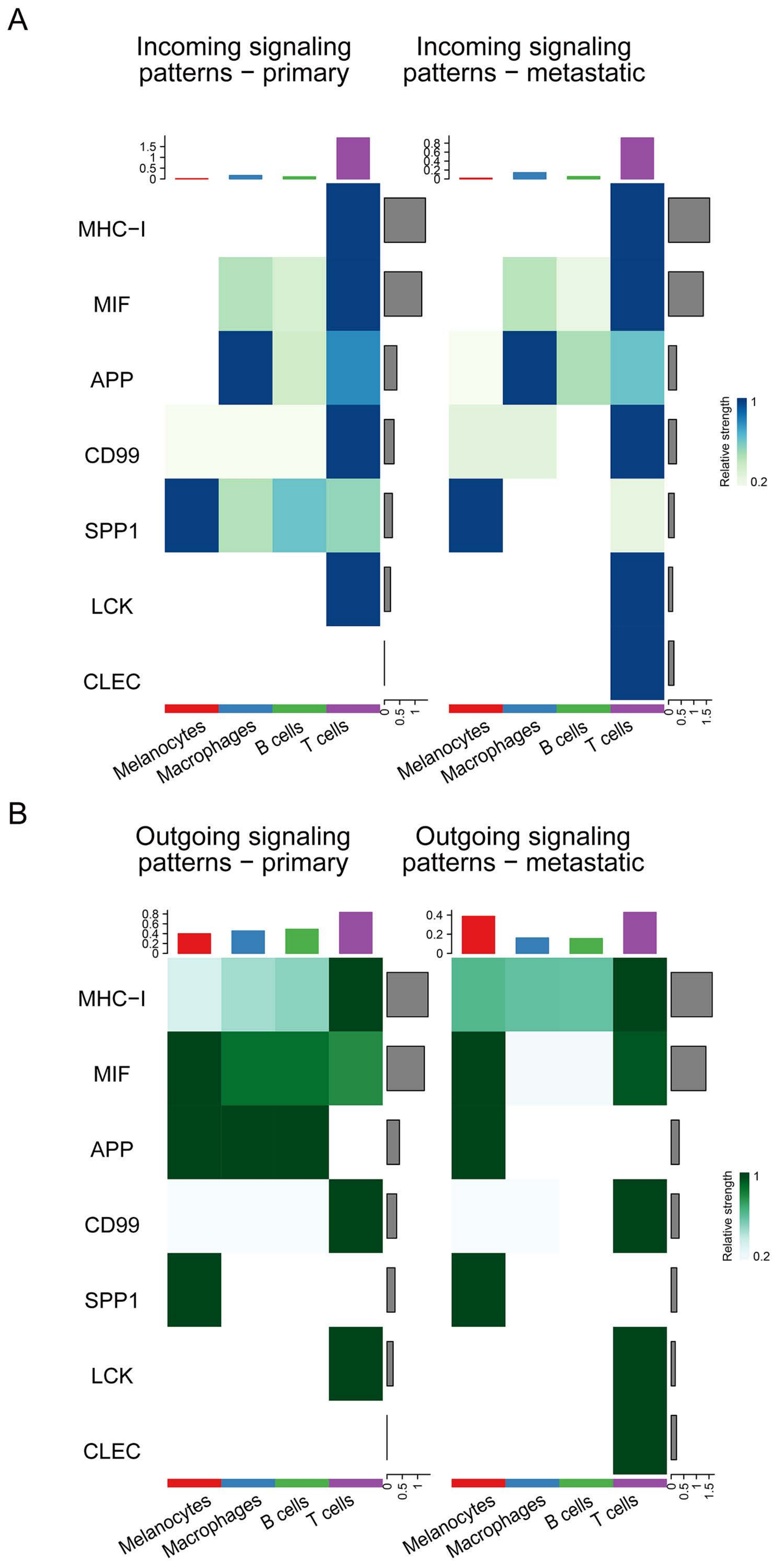
A
Incoming signaling patterns − primary
Incoming signaling patterns − metastatic
MHC−I
MIF
APP
CD99
SPP1
LCK
CLEC
Melanocytes
Macrophages
B cells
T cells
Relative strength
1
0.2
B
Outgoing signaling patterns − primary
Outgoing signaling patterns − metastatic

FigureS4

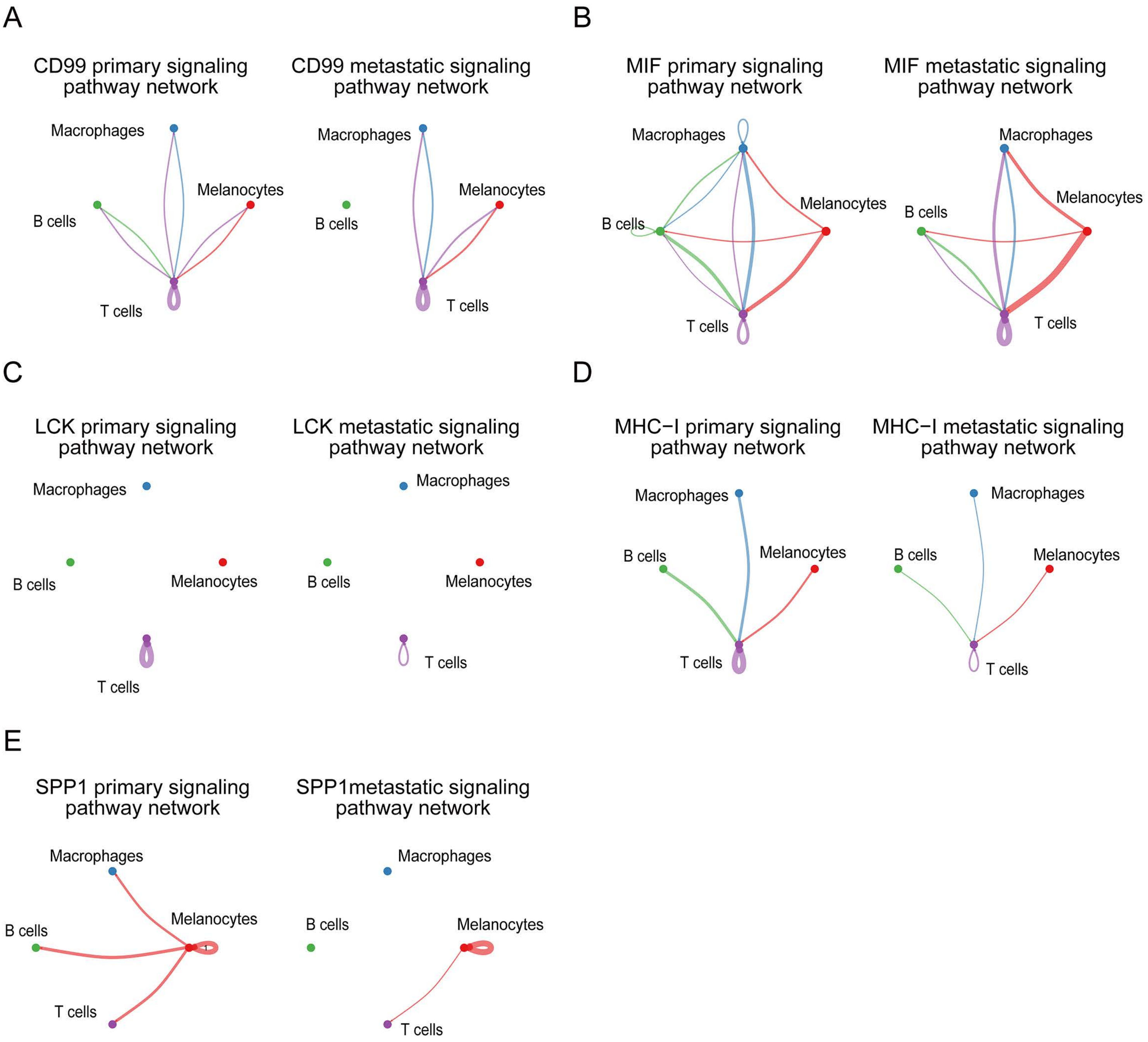
A
CD99 primary signaling pathway network
Macrophages
Melanocytes
B cells
T cells
CD99 metastatic signaling pathway network
Macrophages
Melanocytes
B cells
T cells
B
MIF primary signaling pathway network
Macrophages
Melanocytes
B cells
T cells
MIF metastatic signaling pathway network
Macrophages
Melanocytes
B cells
T cells
C
LCK primary signaling pathway network
Macrophages
B cells
Melanocytes
T cells
LCK metastatic signaling pathway network
Macrophages
B cells
Melanocytes
T cells
D
MHC−I primary signaling pathway network
Macrophages
B cells
Melanocytes
T cells
MHC−I metastatic signaling pathway network
Macrophages
B cells
Melanocytes
T cells
E
SPP1 primary signaling pathway network
Macrophages
Melanocytes
B cells
T cells
SPP1metastatic signaling pathway network
Macrophages
B cells
Melanocytes
T cells

FigureS5

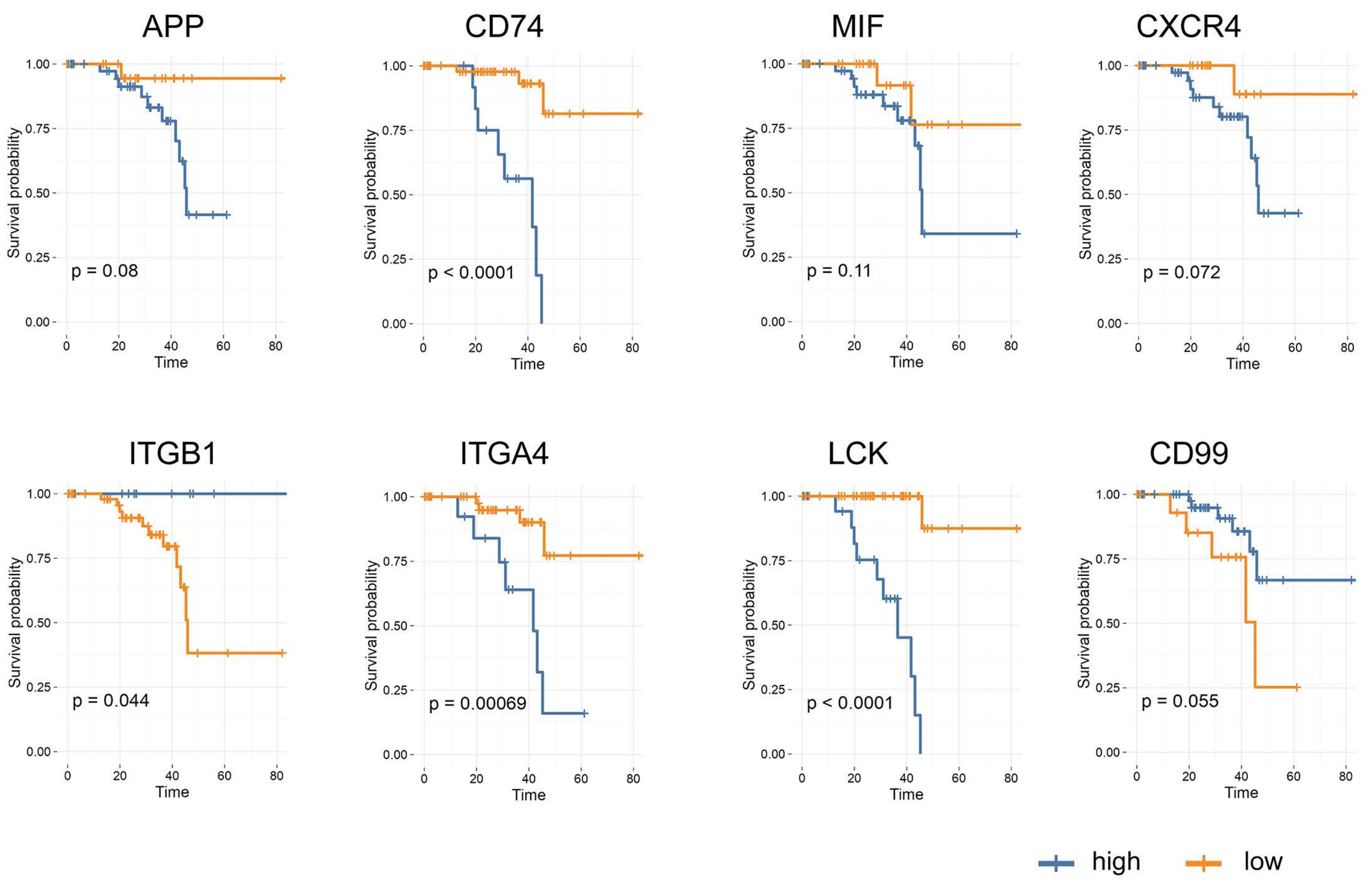
APP
p = 0.08
CD74
p < 0.0001
MIF
p = 0.11
CXCR4
p = 0.072
ITGB1
p = 0.044
ITGA4
p = 0.00069
LCK
p < 0.0001
CD99
p = 0.055
Survival probability
Time
high
low

FigureS6

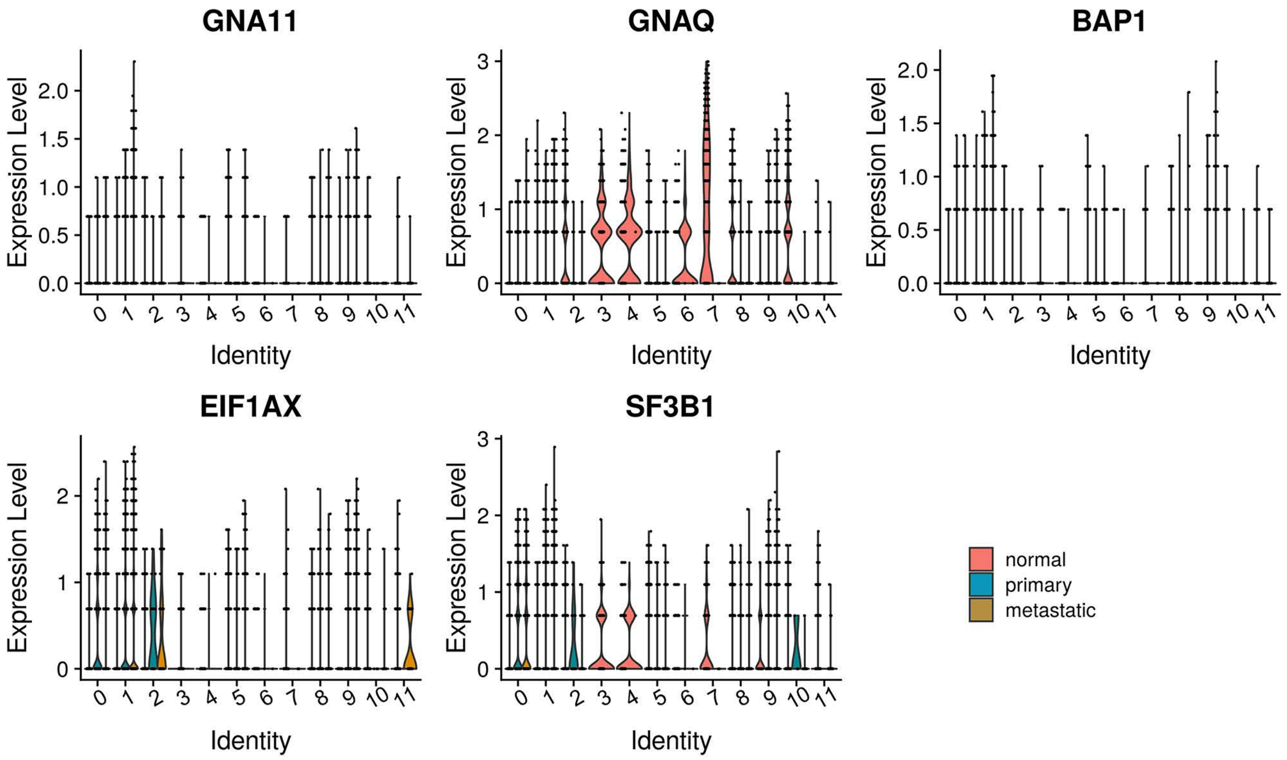
GNA11
GNAQ
BAP1
EIF1AX
SF3B1
Expression Level
Identity
normal
primary
metastatic
